# Simulated diagnostic performance of low-field MRI: Harnessing open-access datasets to evaluate novel devices

**DOI:** 10.1016/j.mri.2021.12.007

**Published:** 2021-12-28

**Authors:** T. Campbell Arnold, Steven N. Baldassano, Brian Litt, Joel M. Stein

**Affiliations:** a Department of Bioengineering, School of Engineering & Applied Science, University of Pennsylvania, Philadelphia, PA 19104, USA; b Center for Neuroengineering and Therapeutics, University of Pennsylvania, Philadelphia, PA 19104, USA; c Department of Neurology, Perelman School of Medicine, University of Pennsylvania, Philadelphia, PA 19104, USA; d Department of Radiology, Perelman School of Medicine, University of Pennsylvania, Philadelphia, PA 19104, USA

**Keywords:** Low-field MRI, Portable MRI, Point-of-care MRI, Simulated clinical trial, Hyperfine, Deep learning

## Abstract

The purpose of this study is to demonstrate a method for virtually evaluating novel imaging devices using machine learning and open-access datasets, here applied to a new, low-field strength portable 64mT MRI device. Paired 3 T and 64mT brain images were used to develop and validate a transformation converting standard clinical images to low-field quality images. Separately, 3 T images were aggregated from open-source databases spanning four neuropathologies: low-grade glioma (LGG, *N* = 76), high-grade glioma (HGG, *N* = 259), stroke (*N* = 28), and multiple sclerosis (MS, *N* = 20). The transformation method was then applied to the open-source data to generate simulated low-field images for each pathology. Convolutional neural networks (DenseNet-121) were trained to detect pathology in axial slices from either 3 T or simulated 64 mT images, and their relative performance was compared to characterize the potential diagnostic capabilities of low-field imaging. Algorithm performance was measured using area under the receiver operating characteristic curve. Across all cohorts, pathology detection was similar between 3 T and simulated 64mT images (LGG: 0.97 vs. 0.98; HGG: 0.96 vs. 0.95; stroke: 0.94 vs. 0.94; MS: 0.90 vs 0.87). Pathology detection was further characterized as a function of lesion size, intensity, and contrast. Simulated images showed decreasing sensitivity for lesions smaller than 4 cm^2^. While simulations cannot replace prospective trials during the evaluation of medical devices, they can provide guidance and justification for prospective studies. Simulated data derived from open-source imaging databases may facilitate testing and validation of new imaging devices.

## 1. Introduction

Modern medical imaging has become a mainstay of optimal patient care, particularly in the diagnosis and management of patients with neurologic disease. While the availability of imaging technology has dramatically increased worldwide in recent decades, the expense and operational complexity of standard imaging systems limits access in underserved areas and developing countries [1]. This so-called “radiology divide” leaves about 90% of the world’s population without access to magnetic resonance imaging (MRI) [2] and almost two-thirds of the population without even basic imaging technology such as ultrasound and X-ray radiography [3–5].

Low-field (LF) strength MRI systems aim to make MRI more accessible, promising lower cost, portability, fewer magnetic field-related safety concerns, and ease of use [6]. Such devices could decrease healthcare expenditures, improve availability in underserved areas, and provide a convenient and ionizing-radiation-free modality for routine or monitoring studies. Portable LF MRI systems may be suitable for hospitalized patients, such as those in intensive care units or isolation wards, for whom transport to a standard clinical scanner carries unacceptable risk [7–10]. More broadly, portable LF MRI units could potentially be used in ambulances, emergency departments, physician’s offices or rural clinics [11,12].

While LF MRI presents clear practical advantages, these systems produce images with lower signal-to-noise, resolution, and tissue contrast compared to their high-field strength (HF) counterparts and are largely designed to complement, and not replace, standard MRI. Prior to deployment for clinical use, the diagnostic capabilities of novel imaging technologies such as portable LF MRI should be evaluated across a wide range of patients and pathologies. The standard approach for device evaluation, improvement, and optimization requires recruiting large numbers of patients and manual image review by radiologists. This process is costly and time-consuming, which can limit the device development cycle. Moreover, selecting target use cases is difficult without basic information about device sensitivity. A complementary approach is to simulate LF images from existing, publicly available HF datasets and leverage machine learning for image interpretation to guide prospective clinical study design. Such datasets, typically compiled for machine learning competitions [13–16] or collaborative research programs [17,18], span broad ranges of pathology and offer a wealth of information for retrospective analysis [19].

Here, we propose a generalizable method for image simulation and interpretation that can guide prospective clinical studies of novel neuroimaging devices. We employ a simple empiric method to transform existing images to a custom domain, converting high-resolution 3 T MR images aggregated from several open-access databases to images matching the resolution and quality of those acquired on a portable 64mT LF MRI scanner. Quantitative measures and manual ratings by radiologists are used to compare image quality between real and simulated 64mT imaging. Separately, convolutional neural networks are trained to detect pathology in axial slices from the HF or simulated LF images, and detection performance is compared between image pairs to characterize the potential diagnostic capabilities of 64mT LF MRI. While automated lesion detection in simulated images does not guarantee detection on actual devices, simulated performance may help indicate whether pursuing a prospective study for a given application is promising. Applied here to LF MRI, this simulated trial approach offers a broadly applicable means for evaluating and optimizing novel medical imaging technologies to complement traditional imaging clinical trials.

## 2. Materials & methods

### 2.1. Data collection

Paired portable 64mT LF (Hyperfine) and same-day standard clinical 3 T HF (Siemens) brain MRI data were collected as part of an ongoing research study approved by the University of Pennsylvania Institutional Review Board. Participants provided informed consent. To develop the domain transformation, we used data from six adult patients with known or suspected hydrocephalus. To validate and assess the generalizability of the domain transformation, we used data from ten adult patients with multiple sclerosis (MS). Fluid-attenuated inversion recovery (FLAIR) images covering the whole brain were collected on each scanner. This sequence was chosen because it 1) is fundamental to clinical imaging for each type of pathology detailed below, 2) provides the most lesion conspicuity across pathologies, 3) is more robust at low field than other sequences such as diffusion-weighted imaging (DWI), and 4) does not rely on exogenous contrast mechanisms. For 64mT imaging, patients received the following 3D fast spin-echo scan optimized for typical brain tissue contrasts (TE = 200 ms, TR = 4 s, TI = 1.4 s, averages = 1, scan time = 9:29 min, resolution = 1.6 × 1.6 × 5 mm). For 3 T imaging, hydrocephalus patients received clinical axial 2D FLAIR imaging with variable sequence parameters (TE = 96–141 ms, TR = 8–10 s, TI = 2.2–2.55 s, resolution = 0.72–0.94 × 0.72–0.94 × 3–6 mm), while MS patients received a standardized 3D FLAIR sequence (TE = 398 ms, TR = 5 s, TI = 1.6 s, averages = 1, scan time = 5:02 min, resolution = 1 mm isotropic).

Separately, retrospective axial FLAIR images obtained at 3 T for a range of pathologies were aggregated from several open-access sources [13–16,20]. Pathologies consisted of high-grade glioma (HGG, *N* = 259), low-grade glioma (LGG, *N* = 76), stroke (*N* = 28), and MS (*N* = 20) [13–16]. Each dataset contained manual segmentations of lesions, which generally manifest as hyperintense areas on FLAIR imaging. These datasets incorporate a range of lesion sizes and signal intensities that can be quantified using the provided lesion segmentations. HGG and LGG lesion segmentations on FLAIR imaging include areas that may represent vasogenic edema or non-enhancing infiltrative neoplasm as well as enhancing components when present. Additionally, non-lesional control scans (*N* = 5) were drawn from the OASIS3 dataset [20]. Table 1 contains information about the different public datasets used in this study. Related web addresses as of publication are listed in section 2.9 Code and data availability.

### 2.2. Domain transformation: high-field to low-field MRI

**Fig. 1.**
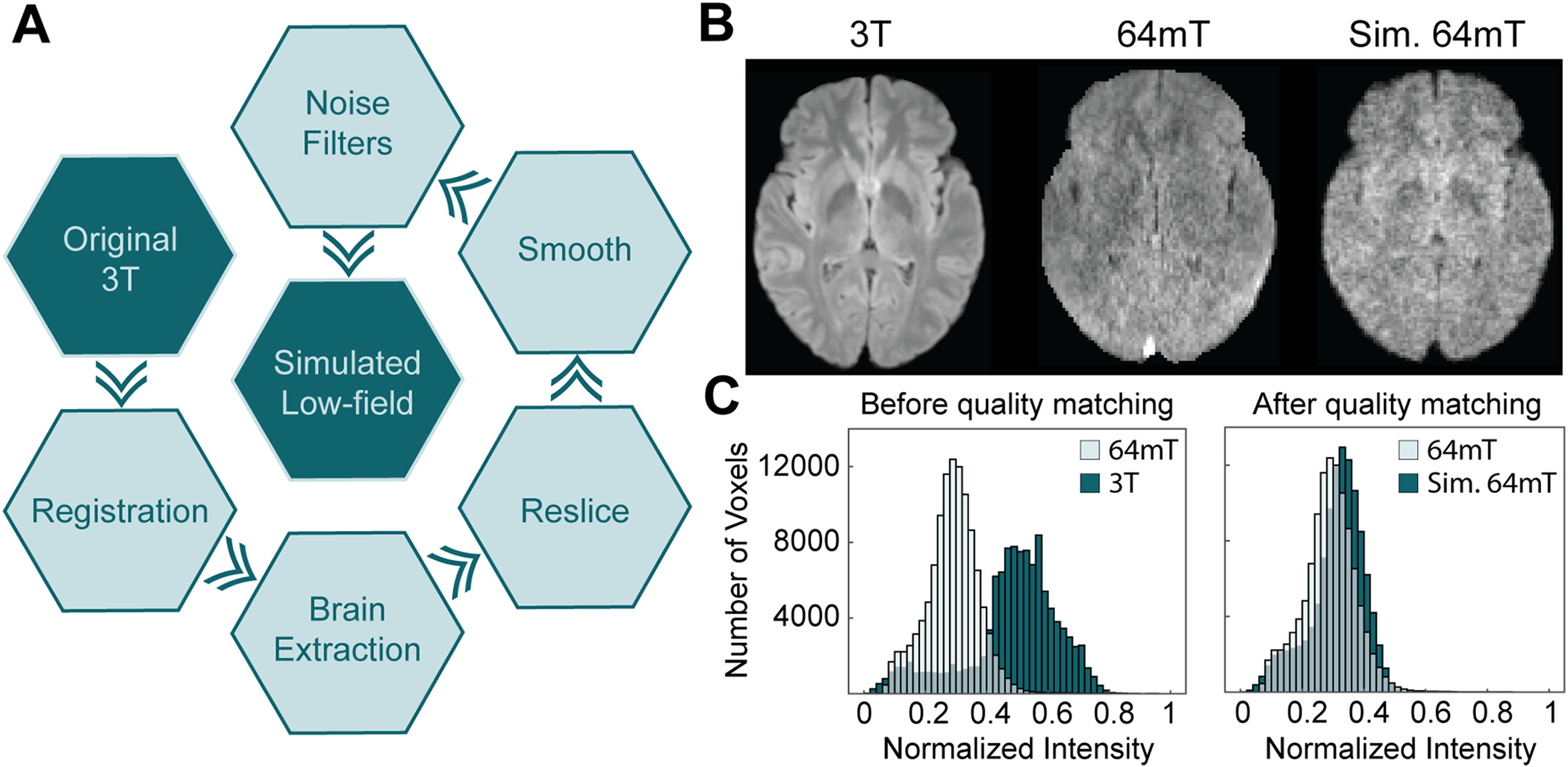
Generating simulated low-field (LF) images. (A) Steps in the image processing pipeline from the original 3 T image (upper-left) to the simulated LF version (center). (B) Example skull-stripped axial FLAR images from a clinical 3 T (left) and a 64mT LF MRI scanner (center). The 3 T image was passed through the image transformation pipeline to produce the simulated LF image (right). (C) To generate simulated LF images resembling actual LF images, histogram features (mean, standard deviation, and skewness) were used to guide image transformation. The intensity histogram distributions relative to actual 64 mT images are shown before (left) and after (right) transformation.

To generate simulated LF images from HF data, we employed a simple image transformation using 3 T and 64mT image pairs from three of the hydrocephalus patients. Transformation steps are listed in Fig. 1A and include registration, brain extraction, re-slicing, Gaussian smoothing, and noise filtering. After registration, brain extraction, and re-slicing simulated images match LF resolution; however, SNR remains substantially higher than actual LF imaging. To better match LF image quality, a series of smoothing kernels and noise filters were applied to simulated images. Noise filter amplitude and smoothing kernel standard deviation were parameterized and fit using training data. First, random noise was smoothed using a 3-D Gaussian kernel with a 0.5 standard deviation. Amplitude of the noise filter was parameterized and added to the image. Next, the image was smoothed using a 3-D Gaussian kernel with a parameterized standard deviation. Finally, an additional noise filter was applied with a parameterized amplitude and smoothing kernel. To determine optimal parameters, we minimized the difference in histogram features between real and simulated images. The objective function consisted of the first three statistical moments (mean, standard deviation, and skewness). An example HF/LF pair and the simulated LF image can be seen in Fig. 1B with the matched intensity histograms shown in Fig. 1C. The transformation was applied to the HF images collected from open-access datasets to produce simulated LF images for gliomas, stroke, and multiple sclerosis.

### 2.3. Domain transformation: quantitative validation

A quantitative validation of the domain transformation was performed using data from three additional hydrocephalus patients. The image transformation method was quantitatively assessed using the gradient entropy, *F*, as a measure of perceived diagnostic image quality. This metric was derived by McGee et al. [21]:

(1) $$F=-\sum_{ij}h_{i,j}log_{2}\left[h_{i,j}\right],$$

(2) $$h_{i,j}=\frac{\left|[1-1]*g_{i,j}\right|}{\sum_{ij}\left|[1-1]*g_{i,j}\right|}.$$

where *g*_*i,j*_ is the pixel value at coordinate *i,j* and * represents the convolution operation. Of 24 metrics evaluated, McGee et al. found gradient entropy (Eq. 1) to have the strongest correlation with radiologists’ perception of image quality in structural MRI. We compared gradient entropy values between 3 T, real 64mT, and simulated 64mT images using a paired *t*-test.

### 2.4. Domain transformation: radiologist validation

**Fig. 2.**
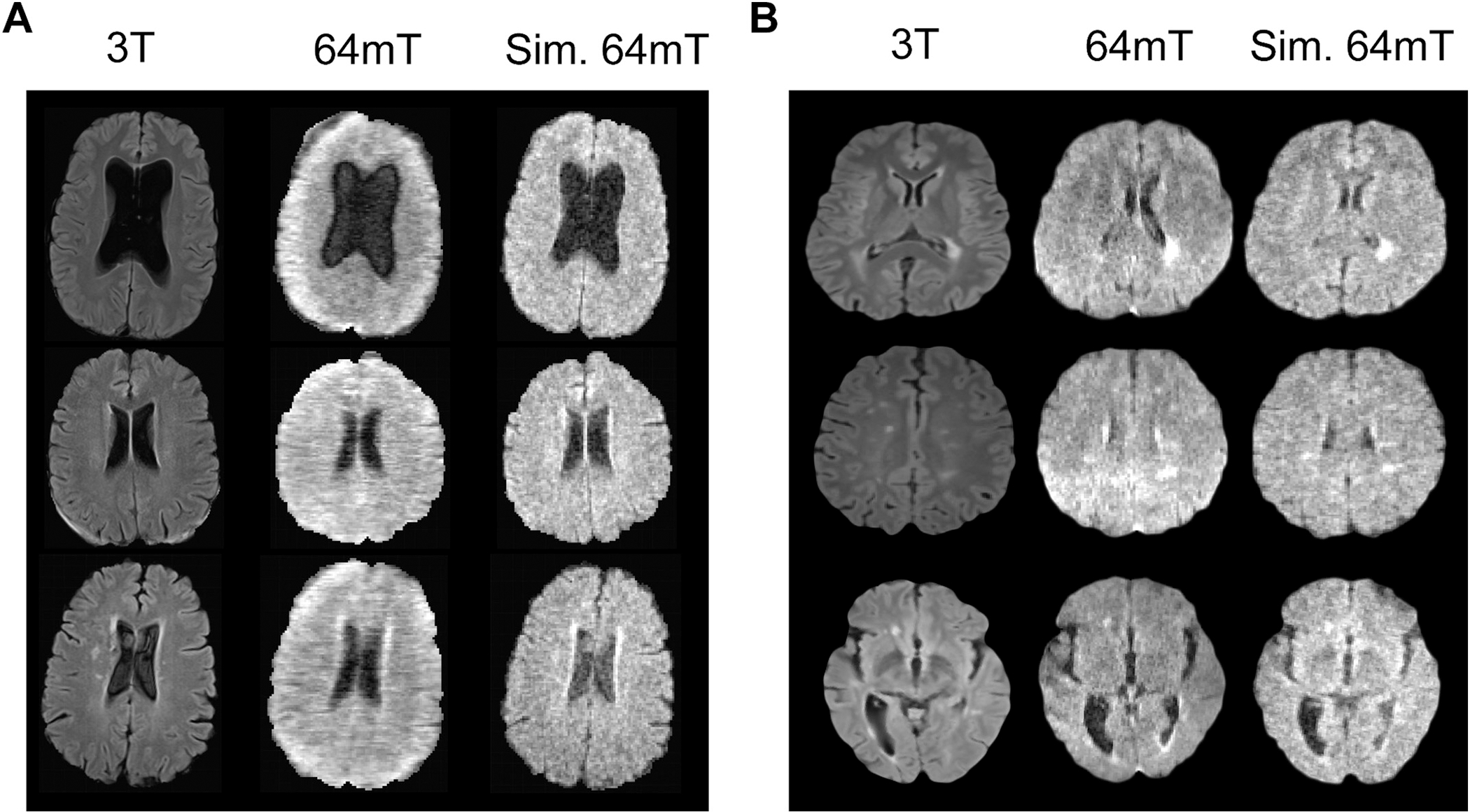
Validation of image transformation method. (A) Transformation applied to three novel hydrocephalus patients not used to develop the domain transformation. 3 T images (column 1), 64mT images (column 2), and simulated 64mT images (column 3). (B) Transformation was applied to ten novel MS patients, three of which are visualized here. No patients visualized were included in the training set used to develop the automated transformation.

For the domain transformation approach to be valid, real and simulated LF images should have similar image quality and interpretability for radiologists. To assess the diagnostic quality of images, we compared ratings from three neuroradiologists (with 4, 5 and 9 years in clinical practice) for real 64mT, simulated 64mT, and 3 T imaging from ten MS patients (Fig. 2B). All imaging was coregistered and paired axial slices were drawn from each image type. We asked the neuroradiologists to rate slices from each image type using a 5-point Likert scale [22]. Each neuroradiologist rated a total of 90 image slices and slice order was randomized. For each slice, neuroradiologists were asked: 1) Do you see white matter lesions in this image? (yes/no), 2) How confident are you in this rating? (1 = excellent, 2 = good, 3 = average, 4 = poor, and 5 = random), 3) What is the diagnostic quality of the image? (1 = excellent, 2 = good, 3 = average, 4 = poor, and 5 = nondiagnostic). Statistical comparisons between 3 T, real 64mT, and simulated 64mT imaging were performed using a paired sample Wilcoxon signed-rank test [22].

### 2.5. Modulating lesion contrast

Differences in magnetic relaxation times and optimal sequence parameters at LF relative to HF can contribute to differences in tissue and lesion contrasts [2]. Although our domain transformation approach does not mathematically model these potential differences, we can use lesion segmentations to evaluate how changes in lesion contrast and conspicuity should be expected to affect detection accuracy. Thus, we prepared additional simulated LF images from the HGG dataset with decreasing signal intensity within the segmented lesions. We chose to run this sub-analysis on the BraTS HGG dataset because the glioma segmentations contained 4 tissue type labels, which allowed us to modulate contrast in each area separately, providing overall better contrast modulation. Lesion intensity values were scaled independently from surrounding brain tissue in 20% increments over a range from 100% (original intensity) to 0% (isointense with background tissue). Isointensity was defined as mean lesion intensity equal to mean intensity of non-lesional tissue in the same slice.

Separate classifiers were trained to identify lesions at each intensity level, allowing for the decoupling of intensity contrast and structural abnormalities in classifier performance. Note that because of structural abnormalities caused by large tumors, such as mass effect, midline shift, and ventricular effacement, as well as intensity heterogeneity within lesions, even isointense lesions may retain some structural and signal alterations after contrast modulation. To minimize the effect of within lesion intensity heterogeneity, we restricted our analysis to patients within the top 50% of lesion homogeneity (*N* = 130), as defined by within tumor signal to noise ratio (SNR). Accurate detection of isointense lesions should therefore be primarily driven by structural abnormalities.

While tissue relaxation rates of pathology remain unknown on the low-field system, this contrast modulation approach can be used to gauge how much contrast would be necessary for lesion detection. Importantly, while we applied this intensity modulation approach to alter contrast between pathology and background tissue, the same approach could be applied to gray matter, white matter, and cerebral spinal fluid segmentations to vary contrast between tissues.

### 2.6. Model architecture

A convolutional neural network model was used to identify pathology in each high-field and simulated low-field dataset. Model construction and training was carried out using the Keras API [23] with TensorFlow [24,25] backend. Model architecture consisted of the DenseNet-121 network [26,27] with initial weights pre-trained on the ImageNet database [28] and four additional densely-connected layers using Xavier initialization. This architecture was consistent across datasets (Supplemental Fig. 1).

### 2.7. Model training

For each dataset, a unique model was trained to perform binary classification of axial slices (lesion present vs. lesion absent). To obtain the best estimate of device sensitivity as well as avoid scanner and site confounds, separate models were trained for each dataset rather than combining datasets and using a multi-class classifier. Separate models were trained on the HF and simulated LF images. Slices were labeled as “lesion present” if at least one pixel from the ground-truth lesion segmentations was present. Each dataset was divided (~9:1 split) into “training” and “test” datasets (Supplemental Table 1). Each patient was confined to either the training or test dataset. Training image order was randomly shuffled. All reported performance metrics were derived from held-out test data. Models were trained for 100 epochs using the Nadam optimizer, a learning rate of 0.002 with decay [29], and a batch size of 32. Batch size was chosen to accommodate VRAM of a Titan X GPU. Training data were augmented using random horizontal flipping. Training hyperparameters were consistent across all models.

### 2.8. Model evaluation

Classification performance was evaluated using two metrics: (1) area under curve (AUC) of the receiver operating characteristic (ROC) and (2) F1 score (harmonic mean of precision and recall). A random chance null model (performance averaged over 1000 trials) was included for comparison. ROC curves were compared using DeLong’s test, implemented using the pROC R package [30]. Logistic regression was used to quantify the impact of lesion size and intensity on detection. Significance of logistic regression parameters was determined by the Wald test.

Class activation maps (CAMs) were generated from shallow and deep convolutional layers to identify discriminative image regions [31]. CAMs were constructed using the output feature map of a given convolutional layer with each feature map channel weighted by the lesion class gradient. Conceptually, CAMs help to interpret model function by visualizing image areas that are driving the model’s classification decision.

In addition to slice-by-slice classification performance, models were evaluated on a per-patient basis. For each test patient, the model assigned a classification score to all axial slices. A sliding convolutional filter was used to determine the mean classification score over several adjacent slices (approximately 1.5 cm in the z-axis) (Supplemental Fig. 2).

### 2.9. Code and data availability

All code related to simulated image generation, classifier design, and statistical analysis can be found at: https://github.com/penn-cnt/Arnold_simulated_clinical_trial. We are grateful to the researchers that published the well-curated, publicly available datasets used in this study. As of publication, these data can be found at MS-SEG 2008 [13]: http://www.ia.unc.edu/MSseg/, BraTS 2019 [14]: https://www.med.upenn.edu/cbica/brats2019.html, MS-SEG 2016 [15]: https://portal.fli-iam.irisa.fr/english-msseg/, ISLES 2015 [16]: http://www.isles-challenge.org/ISLES2015/, OASIS3 [20]: https://www.oasis-brains.org/.

## 3. Results

### 3.1. Image transformation: quantitative validation

Quantitative validation of the image transformation method was performed using data from the three additional hydrocephalus patients not included during the transformation fitting step. For each participant, a standard 3 T FLAIR image was transformed into a simulated LF image for comparison against the authentic LF ground truth. Representative images from each participant are shown in Fig. 2A.

Image quality was quantitatively assessed using the entropy of the MR image gradient. Lower gradient entropy indicates sharper features and correlates strongly with radiologists’ perception of image quality on MRI [21]. Gradient entropy of HF images (mean ± standard deviation: 5.91 ± 0.54) was significantly lower than both the real LF images (7.89 ± 0.90) and simulated LF (7.42 ± 0.73) images (*t*-test, *p* < 0.0001). While there was a statistical difference between the gradient entropy of real and simulated LF images (t-test, *p* < 0.05), the effect size was dramatically reduced compared to the original HF images (0.47 vs 1.98). While perceived quality was modestly higher in simulated LF images, there was substantial overlap of gradient entropy with real LF images, indicating similar image quality between simulated and real images.

### 3.2. Image transformation: radiologist validation

**Fig. 3.**
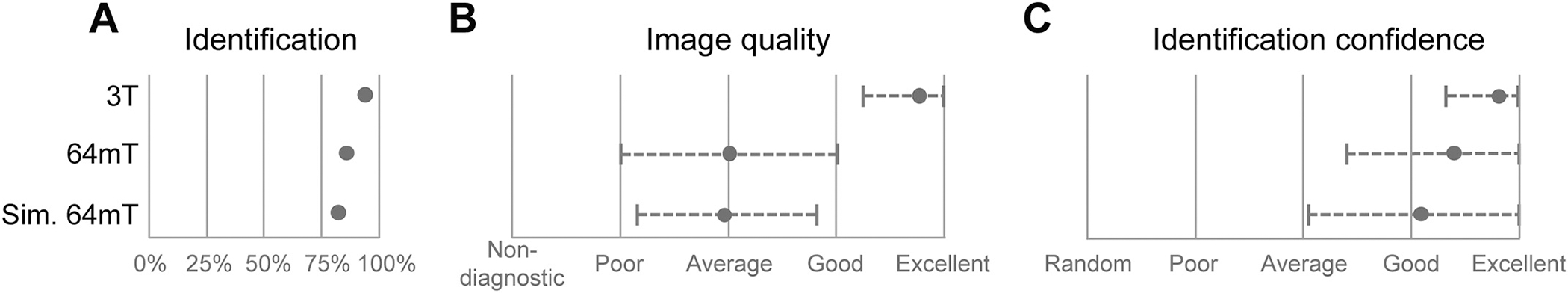
Radiologist ratings of real and simulated images. (A) Radiologists identified lesions at a similar rate in real (86.6%) and simulated (82.2%) 64mT images, both lower than 3 T imaging (94.4%). (B) Image quality was also rated as similar between real and simulated 64mT images (no significant difference, paired sample Wilcoxon signed-rank test, *p* = 0.32). Both real and simulated 64mT images were rated as having significantly lower quality than 3 T imaging (paired sample Wilcoxon signed-rank test, *p* < 0.0001). (C) Raters were however more confident in their ratings for real 64mT compared to simulated images (paired sample Wilcoxon signed-rank test, *p* < 0.01). Confidence ratings of both real and simulated 64mT images were significantly lower than 3 T imaging (paired sample Wilcoxon signed-rank test, p < 0.0001).

Representative image transformations from MS patients are shown in Fig. 2B with additional examples and details in Supplemental Fig. 3. Perceptions of diagnostic quality for real and simulated 64mT images (Fig. 3) were similar (mean ± standard deviation, 2.98 ± 1.04 and 3.04 ± 0.86 respectively) with no statistical difference detected between the ratings (paired sample Wilcoxon signed-rank test, *p* = 0.32). Both real and simulated 64mT images were rated as having average diagnostic quality, which was significantly lower than clinical 3 T imaging, which was rated as having excellent quality (1.27 ± 0.47, paired sample Wilcoxon signed-rank test, *p* < 0.0001). White matter lesions were detected at similar rates in real and simulated 64mT images (86.6% and 82.2% respectively), both lower than the baseline detection rate of 94.4% in 3 T imaging. Confidence in rating the presence or absence of lesions was slightly lower for simulated 64mT (1.88 ± 1.08) compared to real 64mT (1.59 ± 0.96) images (paired sample Wilcoxon signed-rank test, *p* < 0.01). Taken together, these results indicate that radiologists found perceived quality and clinical utility to be similar between real and simulated 64mT imaging.

### 3.3. Comparing pathology detection in standard and simulated low-field images

**Fig. 4.**
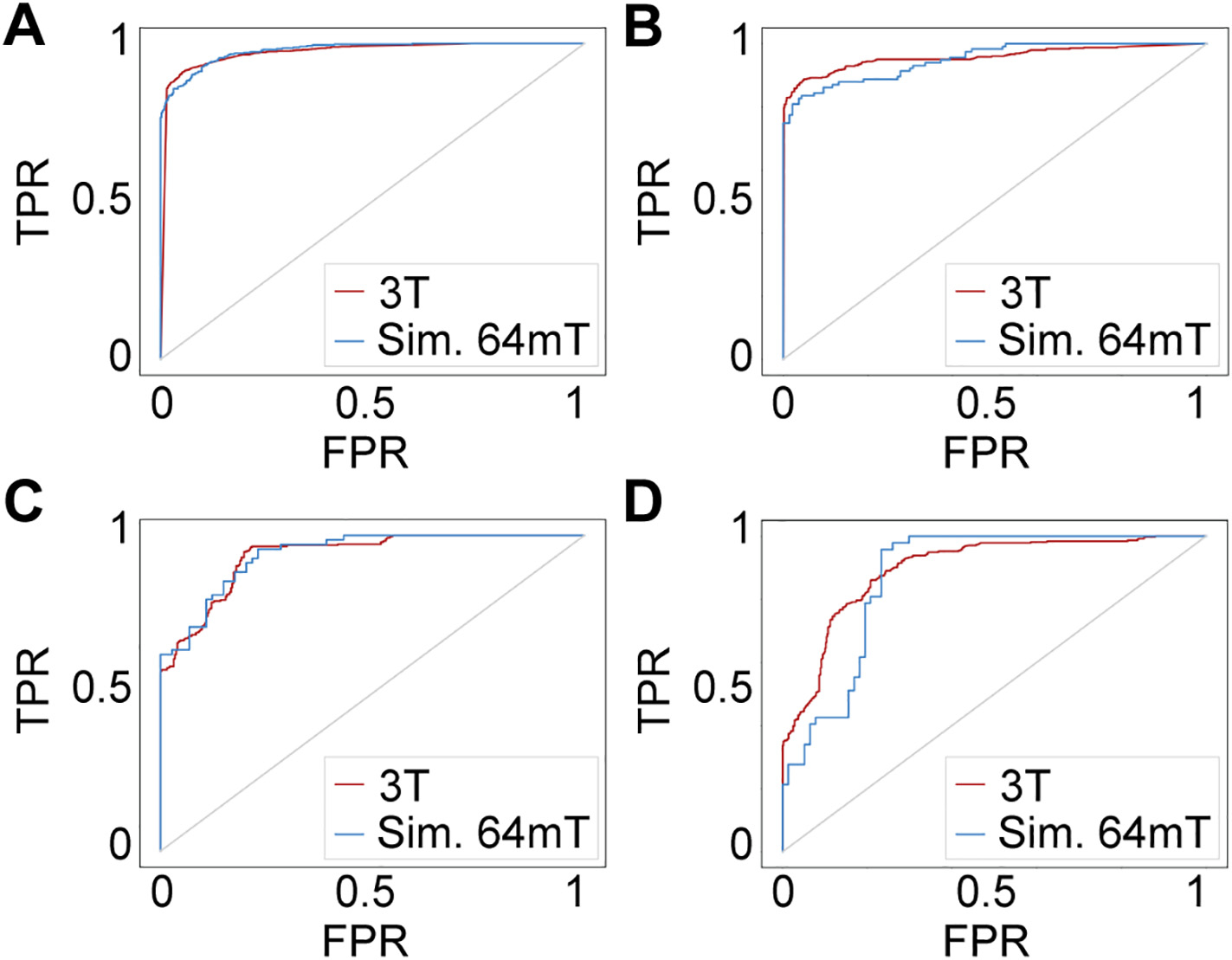
Pathology detection performance. Receiver operating characteristic (ROC) curves shown for binary classification of images with and without pathology. (A) High-grade glioma (*N* = 259) 3 T classifier used 35,883 training images and 3797 testing images, while the simulated 64mT classifier used 8334 training images and 882 testing images. (B) Low-grade glioma (*N* = 76) 3 T classifier used 10,230 training images and 1085 testing images, while the simulated 64mT classifier used 2376 training images and 252 testing images. (C) Ischemic stroke (*N* = 28) 3 T classifier used 2553 training images and 614 testing images, while the simulated 64mT classifier used 865 training images and 143 testing images. (D) Multiple sclerosis (*N* = 20) 3 T classifier used 10,824 training images and 1428 testing images, while the simulated 64mT classifier used 812 training images and 124 testing images. No significant differences between 3 T and simulated 64mT ROC curves were detected for any pathology. Abbreviations: True positive rate (TPR), False positive rate (FPR), Simulated (Sim.).

Deep learning models were trained to perform binary classification of images in each disease cohort using either HF or simulated LF MRI. ROC curves for each cohort are shown in Fig. 4. Despite significant image degradation, per-slice classifier performance was similar for HF and simulated LF MRI across all pathologies (Table 2). As expected, accuracy on more subtle pathology (MS lesions) was lower than more prominent pathology for both HF and simulated LF MRI datasets. The performance achieved is comparable to previous benchmarks using deep learning for detection of brain masses [32,33], and MS lesions [34], and significantly exceeded null models in all cohorts tested.

### 3.4. Characterizing low-field pathology detection

**Fig. 5.**
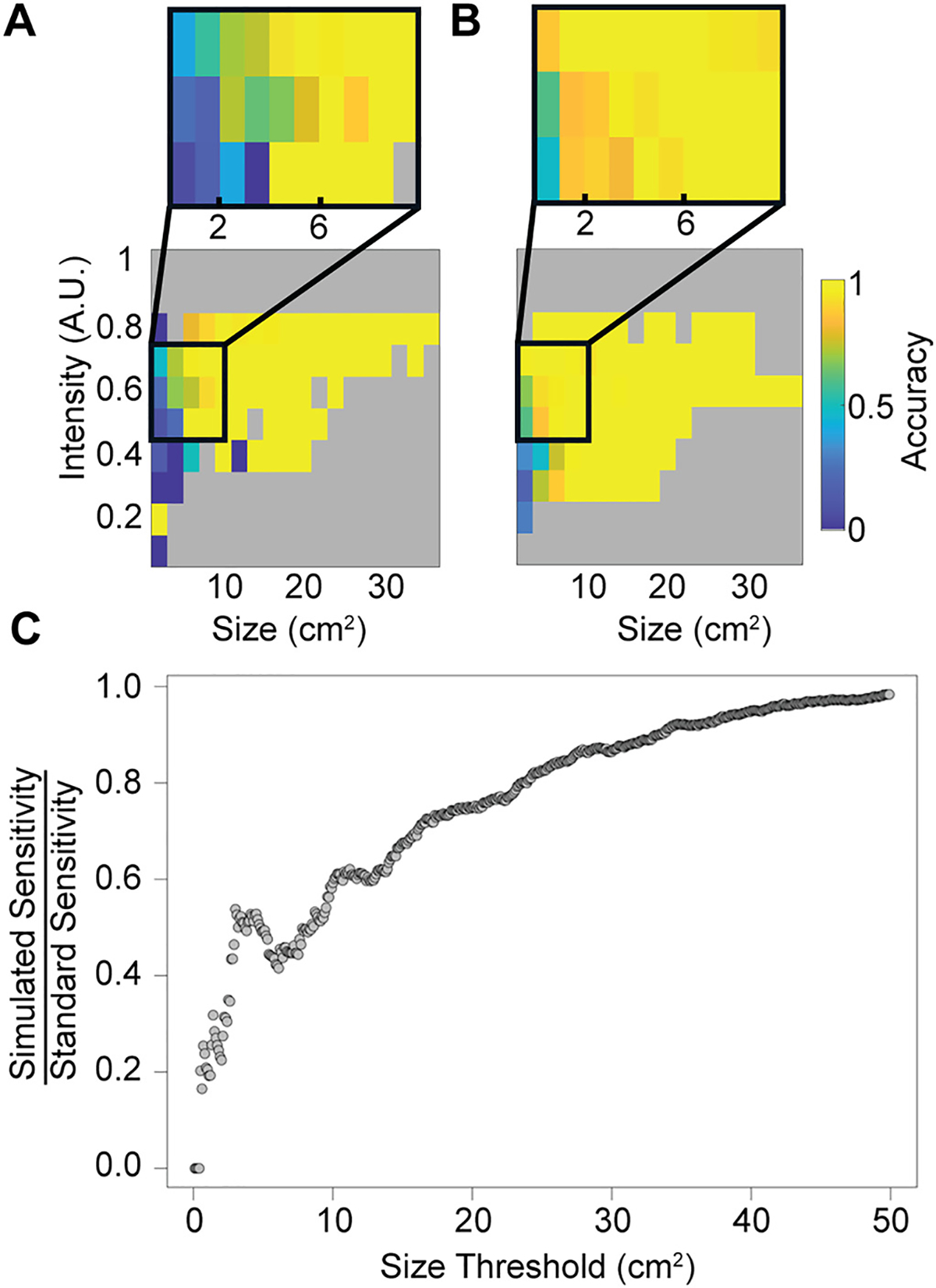
Detection sensitivity as a function of lesion size and scaled intensity. Sensitivity of the deep learning classifiers for detecting lesions in the test set is shown in the (A) simulated LF (1401 testing slices) and (B) standard HF (6924 testing slices) images. Areas highlighting discrepancies between the datasets are highlighted in image insets. (C) Sensitivity of lesion detection in simulated LF images relative to HF images. Each point represents the sensitivity ratio measured on all lesions smaller than the given size threshold. Note that sensitivity is similar between image types when averaged over all lesions but differs significantly when restricted to smaller lesions.

To broadly characterize pathology detection capabilities in simulated LF images, detection sensitivity was aggregated across all pathologies and modeled using a logistic regression as a function of lesion size and intensity as shown in Fig. 5A & 5B. For both HF and simulated LF images, sensitivity was more strongly associated with lesion size, though both parameters reached statistical significance (standard: z_size_ = 13.5, p_size_ < 2e-16, z_intensity_ = 9.2, p_intensity_ < 2e-16; simulated low-field: z_size_ = 8.5, p_size_ < 2e-16, z_intensity_ = 3.9, p_intensity_ < 9e-5). HF imaging outperformed simulated LF imaging for detection of smaller or less intense lesions as shown in Fig. 5C. While performance did not vary significantly between HF and simulated LF images across the cohorts as a whole, sensitivity differences in this subgroup analysis suggests a performance drop-off when using LF imaging for 1–4 cm^2^ lesions (Fig. 5A & 5B). These findings are agnostic to pathology type and may serve as generalizable performance thresholds for FLAIR at 64mT in yet-untested patient populations.

### 3.5. Patient-level classification

**Fig. 6.**
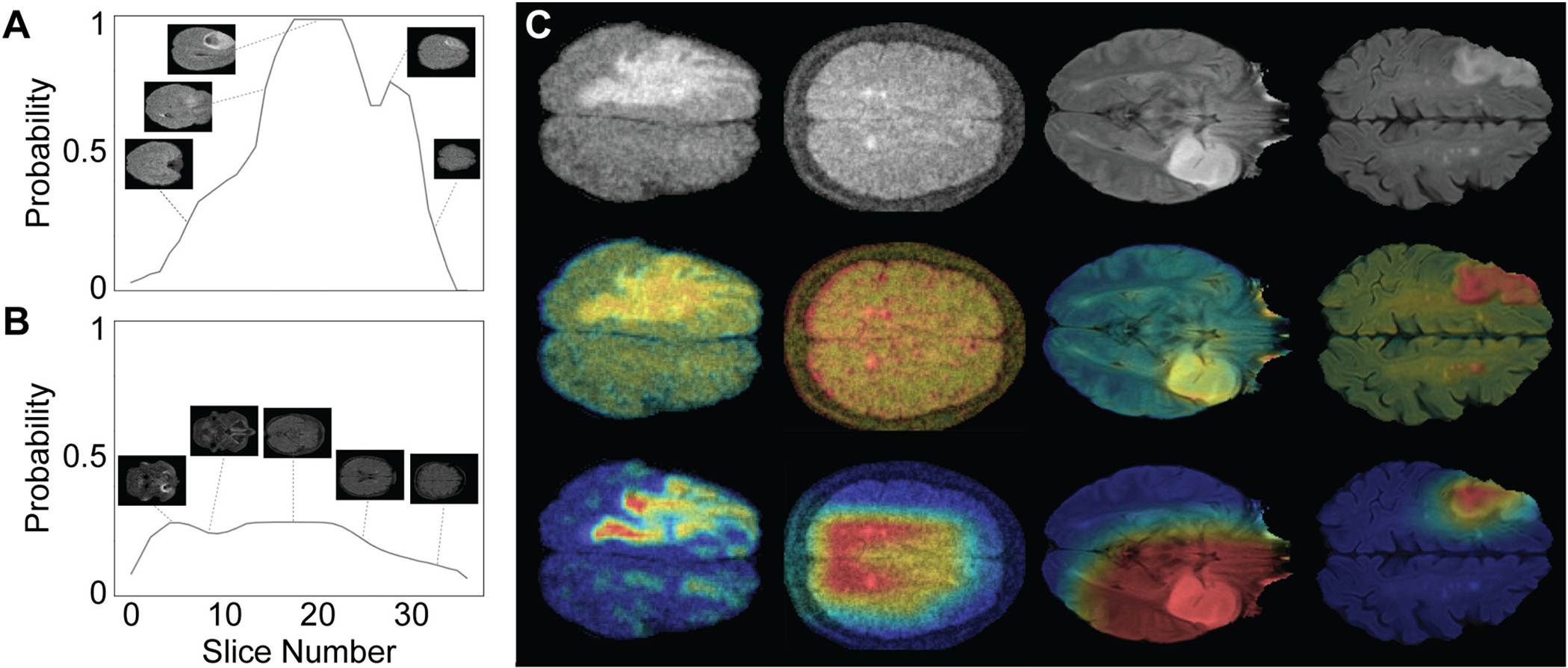
Model validation and interpretability. Panel A and B provide examples of per-patient pathology detection. Convolutional filters were used to generate average lesion probability values across several adjacent axial slices. A threshold value for patient-level classification was determined empirically by maximizing per-patient classification accuracy in the training set. Sample plots are shown for a patient with HGG (A) and a control patient (B) using simulated low-field imaging. (C) Class activation mapping. Row 1: Sample images of high-grade glioma (low-field), multiple sclerosis (low-field), low-grade glioma (standard), and ischemic stroke (standard). Row 2: CAMs generated from shallow network layer for each pathology. Row 3: CAMs generated from deepest convolutional network layer for each pathology.

In addition to per-slice performance, we assessed pathology detection on a per-patient basis. Algorithms were evaluated over 17 held-out patients (three MS, four stroke, five HGG, five LGG) and achieved 100% sensitivity in both HF and simulated LF images. The LGG classifier was also evaluated using five control subjects, and correctly identified all subjects as non-lesional (100% specificity in both HF and simulated LF images) as shown in Fig. 6A & B.

### 3.6. Class activation mapping

We used class activation mapping to probe which image regions were driving algorithm decisions as shown in Fig. 6C. As expected, areas containing pathology are the primary drivers of classification at both shallow and deep layers. The deep CAM for the MS model also reveals that this model attends to periventricular areas known to be clinically important for lesion identification. These findings are reassuring that the tested models are detecting pathology of interest as expected and may have empirically captured features of the typical disease distribution. It is important to note that CAMs serve as an approximate representation of model attention, and each convolutional model layer has a unique CAM. While interpretation of CAMs alone is difficult due to the nonlinear nature of neural networks, the CAMs and the patient level visualizations provide convergent evidence that our models are attending to pathological features.

### 3.7. Determining the effect of lesion intensity on detection

Again, a potential limitation of the simulated image generation method is that it does not account for possible changes in LF lesion to background tissue contrast relationships due to differences in relaxation times or pulse sequences. Here, we quantify detection robustness by measuring performance over a range of lesion contrasts (outlined in *2.3. Modulating lesion contrast*) for HGG images. This approach also allows us to assess the relative importance of lesion to background tissue contrast in comparison to structural distortion from large brain tumors.

**Fig. 7.**
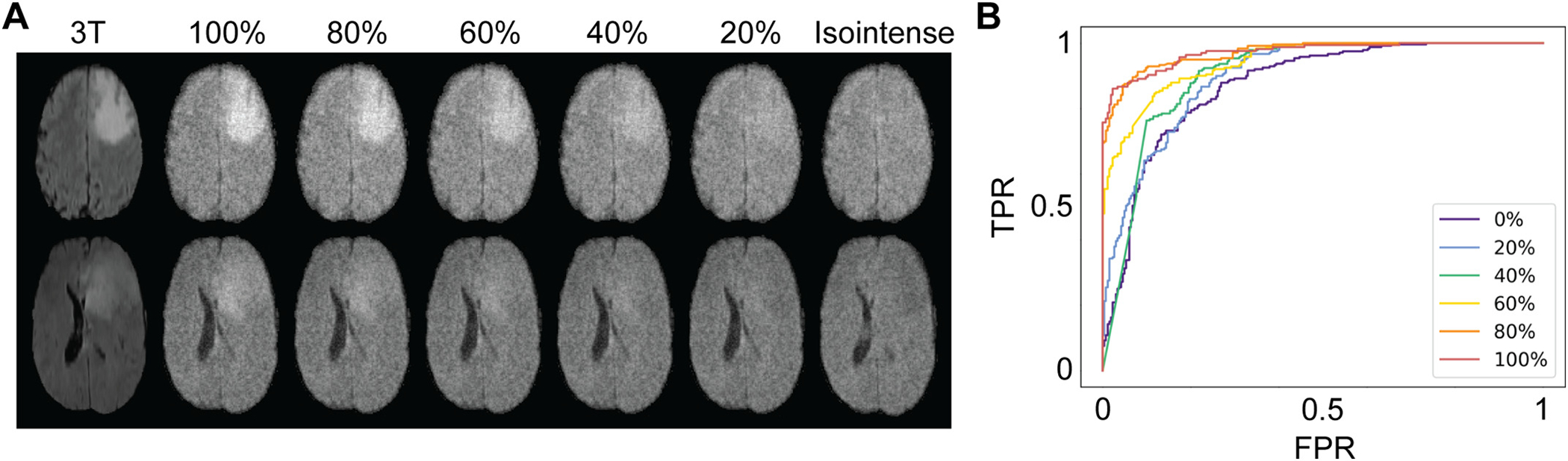
Intensity modulation to explore effects of intensity contrast. (A) Intensity values of the pathology segmentation were modulated over a range from normal intensity (100%) to isointense (tumor = background). (B) For each image subset, a classifier was trained to distinguish pathological and normal slices (N = 259, 8334 training slices, 882 testing slices). AUC varied directly with lesion contrast but remained significantly better than chance even in the isointense cohort, which likely reflects structural deformations (such as ventricular effacement and midline shift as in the bottom row of panel A) or residual signal intensity heterogeneity.

ROC curves for detection of HGGs are shown in Fig. 7. Compared to detection of full-intensity tumors (AUC = 0.972), there was a statistically significant decrease in performance for tumors with relative contrast of 60% or less (AUC_80_ = 0.972, p = NS; AUC_60_ = 0.943, *p* = 0.01; AUC_40_ = 0.905, *p* = 2.0e-5; AUC_20_ = 0.901, *p* = 2.2e-6; AUC_0_ = 0.874, *p* = 1.7e-8). While contrast had a substantial impact on lesion detection, an AUC of 0.874 and F1 of 0.71 was achieved even for isointense lesions, indicating that in this dataset even with reduced lesion contrast large pathology could be identified due to structural deformation.

## 4. Discussion

In this study we propose a generalizable method of image simulation and interpretation for the assessment of novel imaging modalities, particularly those that involve tradeoffs and lower image quality compared to an accepted standard, applied here to portable LF MRI. By leveraging open-access datasets, this virtual trial paradigm permits rapid, low-cost assessment of a device’s potential diagnostic capabilities across a range of pathologies with limited real device data. We assert that this approach can help to address challenges in medical device development, regulatory approval, and clinical trial design.

Portable LF MRI offers an exciting opportunity for improving imaging accessibility in low-resource environments and enables point-of-care MRI. These scanners have relatively low manufacturing and operating costs and may help stem the increasing contribution of medical imaging to healthcare expenditures [35]. To accelerate device development cycles and reduce the cost of bringing devices to market, it is pivotal that tools are developed to allow rapid prototyping, efficient regulatory approval, and expedited deployment. Virtual clinical trials can efficiently guide medical imaging technology evaluation by simulating patients, imaging systems, and image interpreters [36]. For example, in breast tomography, Barufaldi et al. developed a virtual breast phantom and analytical pipeline that can simulate a clinical trial for several hundred patients per day [37]. While not a replacement for traditional prospective studies, simulated clinical trials may offer significant value as the FDA considers devices prior to extensive prospective data collection. Simulated trials could contribute to early feasibility studies (EFS) or provide supporting evidence for investigational device exemption (IDE) approval. Additionally, simulated trials could identify key patient populations or indications to prioritize for standard clinical trials involving imaging. When considering a particular disease process, a simulated trial approach could help to establish benchmarks that a proposed device (such as a scanner or pulse sequence) must meet to provide sufficient diagnostic performance.

Based on simulated LF images, our study suggests LF MRI scanners should detect many brain lesions with comparable performance to standard MR imaging. However, simulated LF imaging was sensitive to lesion size. Accuracy was lower for 1–4 cm^2^ lesions as shown in Fig. 5A. These findings indicate that LF MRI may perform adequately for identifying macro-scale pathology (most gliomas, medium-large vessel stroke, etc.) or measuring major brain structures, but may be less reliable for more subtle pathologies (small MS lesions, embolic infarcts).

We expect portable LF MRI to be used predominantly for clinical applications where standard MRI is either not feasible or delayed. For this reason and as a proof of concept, our analysis is limited to basic diagnostic capability (i.e. lesion sensitivity), defining a range of expected size and signal intensity thresholds, rather than more complex image interpretation such as distinguishing among pathologies, precise lesion segmentation, or tracking lesion evolution over time. While we compare performance of LF MRI to 3 T MR devices, the practical alternative in certain use cases (ICU patients, underserved communities, in-office disease tracking) would be portable CT scans or no imaging at all. In these settings, LF MRI may have advantages over CT such as increased tissue contrast and lack of ionizing radiation exposure.

This work underscores the power of open-access clinical databases to facilitate translational research. Platforms for data sharing, such as XNAT Central [18], iEEG Portal [38], and crowdsourced competitions [39] have led to rapid advances in machine learning. While public databases provide diverse repositories of patient data with sufficient sample sizes to train deep learning algorithms, most medical imaging data remains federated across institutions [40]. Further data-sharing efforts designed explicitly for evaluating devices and software for regulatory approval could reduce the cost and time necessary to bring innovative imaging technology to the clinic. Recently radiology has shifted toward centralizing algorithms while maintaining individual data ownership [41]. While this approach may facilitate algorithm validation across research groups, it precludes creative use of multi-institutional data for applications beyond algorithm testing.

The simulated trial paradigm presented here is meant to serve as a framework for applying pre-existing datasets and deep learning to explore the expected performance of novel diagnostic devices. However, the approach brings with it several important limitations and methodological considerations. Most importantly, the utility of simulated data is directly linked to the transformation method quality. Here, we implemented a relatively simple histogram matching based algorithm. While the present method approximates SNR and resolution in LF images, it does not account for other potential field strength, pulse sequence and device-specific artifacts that may affect lesion conspicuity and image quality.

T1 and T2* relaxation times vary as a function of magnetic field strength, which impacts intensity and contrast between tissues at low-field [2]. Additionally, eddy currents and permanent magnet imperfections cause device specific artifacts. Neither tissue contrast differences nor image artifacts are modeled in our current approach. More advanced transformation methods, including generative adversarial networks (GANs), synthetic MRI, and other quantitative methods [42–45] could improve simulation quality and potentially overcome some of these limitations of our present approach.

However, transformation algorithms that learn by example, such as GANs, require large amounts of data. Methods that simulate images with a low N can be advantageous in certain situations [44]. Specifically, low-data requirement methods can be useful for evaluating new devices or during the prototyping process, where available data are scarce. While data-driven methods may produce closer matched simulations in the long run, these methods may not be feasible for all applications. Importantly, the simulated trial approach is not meant to serve as a replacement for prospective clinical trials. While more advanced methods such as GANs may improve image-to-image simulation quality, there will still likely be a gap between real and simulated images, especially in patients with pathology [45]. Simulations can provide useful guidance, including expected outcomes for prospective trials, however these retrospective analyses cannot provide the same level of scientific evidence as prospective clinical trials.

The scope of the present work is also limited to a specific field strength and pulse sequence. We only applied the domain transformation to 3 T FLAIR imaging with the goal of simulating 64mT data. Image quality at low-field varies significantly depending on the pulse sequence, which means domain transformation results are unlikely to generalize across sequences. For instance, DWI is particularly susceptible to artifacts due to distortions, eddy current, and system stability. While more complex simulations incorporating multiple pulse sequences could be developed using the current framework, domain transformations would need to be developed for each sequence independently and it may be difficult to accurately simulate sequences that are significantly impacted by image artifacts. Additionally, our study only evaluated the domain transformation approach for 3 T to 64mT. To validate that the approach is more broadly applicable, it should be evaluated across a range of field strengths such as 7 T to 3 T or 3 T to 1.5 T [46].

Additionally, special consideration should be taken when interpreting the machine learning results that compare 3 T and simulated 64mT datasets. While performance of pathology detection was similar between datasets, pathology detection is only one component of diagnostic imaging, and these results should not be interpreted as equivalent clinical utility between the devices. Furthermore, our approach is limited to a single sequence and performs a simple detection task using separate classifiers for each pathology. Radiologists use multiple contrasts and incorporate patient information when making a clinical diagnosis. Additionally, while our approach likely provides the best estimate of device sensitivity and avoids potential scanner and site confounds associated with combining publicly available datasets, it does not provide insight into device specificity for distinguishing different pathologies. In future work, it may be possible to reconcile scanner and site differences using data harmonization methods [47–49].

## 5. Conclusion

In this study, we have proposed a method for guiding evaluation of imaging devices via simulated trials, incorporating domain transfer and automated pathology detection, and demonstrated its application to a new portable LF MRI device. This method allows for rapid evaluation of actual or proposed diagnostic imaging devices and can provide guidance and justification for prospective studies. In our simulations, we found that gliomas, strokes, and multiple sclerosis lesions could be detected in LF quality images and characterized size and signal intensity differences affecting lesion detection. This work additionally highlights the importance of centralized data sharing for device design and validation.

## Supplementary Material

1

## Figures and Tables

**Table 1:** List of open-access neuropathology datasets used in this study.

Dataset	BraTS 2019	ISLES 2015	MICCAI 2008	MS-SEG-2016

Pathology	Glioma (HGG & LGG)	Stroke	Multiple Sclerosis (MS)	Multiple Sclerosis (MS)
Patients	335 (259 HGG, 76 LGG)	28	10	10
Center/Scanners	19	2	2	3
Classes	3 (enhanced, non-enhanced, edema)	1 (infarct)	1 (MS lesion)	1 (MS lesion)

Abbreviations: High Grade Glioma (HGG), Low Grade Glioma (LGG), Multiple Sclerosis (MS).

**Table 2:** Performance metrics for each pathology type using standard or simulated low-field images.

Pathology	Standard AUC	Low-Field AUC	P_AUC_	Standard F1	Low-Field F1	Null Model F1

HGG	0.972	0.978	0.16	0.920	0.896	0.481 ± 0.01
LGG	0.957	0.949	0.61	0.885	0.880	0.468 ± 0.02
Stroke	0.936	0.940	0.83	0.772	0.761	0.485 ± 0.02
MS	0.896	0.873	0.49	0.766	0.745	0.442 ± 0.02

Abbreviations: High Grade Glioma (HGG), Low Grade Glioma (LGG), Multiple Sclerosis (MS), Area Under the Curve (AUC).

## Abbreviations:

LF: Low-field strength
HF: high-field strength
LGG: low-grade glioma
HGG: high-grade glioma
MS: multiple sclerosis
FLAIR: fluid-attenuated inversion recovery
SNR: signal to noise ratio
AUC: area under curve
ROC: receiver operating characteristic
CAMs: class activation maps
EFS: early feasibility studies
IDE: investigational device exemption

## References

[1] Ogbole GI, Adeyomoye AO, Badu-Peprah A, Mensah Y, Nzeh DA. Survey of magnetic resonance imaging availability in West Africa. Pan Afr Med J 2018;30. 10.11604/pamj.2018.30.240.14000. PMC6295297 30574259

[2] Marques JP, Simonis FFJ, Webb AG. Low-field MRI: an MR physics perspective. J Magn Reson Imaging 2019;49:1528–42. 10.1002/jmri.26637. 30637943 PMC6590434

[3] Mollura DJ, Shah N, Mazal J. White paper report of the 2013 RAD-AID conference: I. J Am Coll Radiol 2014;11:913–9. 10.1016/j.jacr.2014.03.026. 25189930

[4] Mollura D, Lungren MP. Radiology in global health. New York, NY: Springer; 2014.

[5] Maru DS-R, Schwarz R, Andrews J, Basu S, Sharma A, Moore C. Turning a blind eye: the mobilization of radiology services in resource-poor regions. Glob Health 2010;6:18. 10.1186/1744-8603-6-18. PMC2964530 20946643

[6] Campbell-Washburn AE, Ramasawmy R, Restivo MC, Bhattacharya I, Basar B, Herzka DA, Opportunities in interventional and diagnostic imaging by using high-performance low-field-strength MRI. Radiology 2019;293:384–93. 10.1148/radiol.2019190452. 31573398 PMC6823617

[7] Sheth KN, Mazurek MH, Yuen MM, Cahn BA, Shah JT, Ward A, Assessment of brain injury using portable, low-field magnetic resonance imaging at the bedside of critically ill patients. JAMA Neurol 2020. 10.1001/jamaneurol.2020.3263. PMC7489395 32897296

[8] Mazurek MH, Cahn BA, Yuen MM, Prabhat AM, Chavva IR, Shah JT, Portable, bedside, low-field magnetic resonance imaging for evaluation of intracerebral hemorrhage. Nat Commun 2021;12:5119. 10.1038/s41467-021-25441-6. 34433813 PMC8387402

[9] Turpin J, Unadkat P, Thomas J, Kleiner N, Khazanehdari S, Wanchoo S, Portable magnetic resonance imaging for ICU patients. Crit Care Explor 2020;2: e0306. 10.1097/cce.0000000000000306. 33381764 PMC7769347

[10] Heiss R, Grodzki DM, Horger W, Uder M, Nagel AM, Bickelhaupt S. High-performance low field MRI enables visualization of persistent pulmonary damage after COVID-19. Magn Reson Imaging 2021;76:49–51. 10.1016/j.mri.2020.11.004. 33220447 PMC7673210

[11] Deoni SCL, Bruchhage MMK, Beauchemin J, Volpe A, D’Sa V, Huentelman M, Accessible pediatric neuroimaging using a low field strength MRI scanner. Neuroimage 2021;238:118273. 10.1016/j.neuroimage.2021.118273. 34146712

[12] Shen FX, Wolf SM, Bhavnani S, Deoni S, Elison JT, Fair D, Emerging ethical issues raised by highly portable MRI research in remote and resource-limited international settings. Neuroimage 2021;118210. 10.1016/j.neuroimage.2021.118210. 34062266 PMC8382487

[13] Styner M, Lee J, Chin B, Chin MS, Commowick O, Tran H, 3D segmentation in the clinic: A grand challenge II: MS lesion segmentation. MIDAS journal 2008;1: 1–6.

[14] Bakas S, Reyes M, Jakab A, Bauer S, Rempfler M, Crimi A, Identifying the best machine learning algorithms for brain tumor segmentation, progression assessment, and overall survival prediction in the BRATS. arXiv preprint 2018: arXiv:1811.02629.

[15] Commowick O, Istace A, Kain M, Laurent B, Leray F, Simon M, Objective evaluation of multiple sclerosis lesion segmentation using a data management and processing infrastructure. Sci Rep 2018;8. 10.1038/s41598-018-31911-7. 30209345 PMC6135867

[16] Maier O, Menze BH, von der Gablentz J, Häni L, Heinrich MP, Liebrand M, ISLES 2015 - a public evaluation benchmark for ischemic stroke lesion segmentation from multispectral MRI. Med Image Anal 2017;35:250–69. 10.1016/j.media.2016.07.009. 27475911 PMC5099118

[17] Allen B, Seltzer SE, Langlotz CP, Dreyer KP, Summers RM, Petrick N, A road map for translational research on artificial intelligence in medical imaging: from the 2018 National Institutes of Health/RSNA/ACR/the academy workshop. J Am Coll Radiol 2019;16:1179–89. 10.1016/j.jacr.2019.04.014. 31151893

[18] Herrick R, Horton W, Olsen T, McKay M, Archie KA, Marcus DS. XNAT central: open sourcing imaging research data. Neuroimage 2016;124:1093–6. 10.1016/j.neuroimage.2015.06.076. 26143202 PMC4965359

[19] Prevedello LM, Halabi SS, Shih G, Wu CC, Kohli MD, Chokshi FH, Challenges related to artificial intelligence research in medical imaging and the importance of image analysis competitions. Radiol Artif Intell 2019;1:e180031. 10.1148/ryai.2019180031. 33937783 PMC8017381

[20] LaMontagne PJ, Benzinger TLS, Morris JC, Keefe S, Hornbeck R, Xiong C, OASIS-3: Longitudinal neuroimaging, clinical, and cognitive dataset for normal aging and Alzheimer Disease. MedRxiv 2019. 10.1101/2019.12.13.19014902.

[21] McGee KP, Manduca A, Felmlee JP, Riederer SJ, Ehman RL. Image metric-based correction (autocorrection) of motion effects: analysis of image metrics. J Magn Reson Imaging 2000;11:174–81. 10.1002/(sici)1522-2586(200002)11:2&lt;174::aid-jmri15&gt;3.0.co;2-3. 10713951

[22] Chandarana H, Bagga B, Huang C, Dane B, Petrocelli R, Bruno M, Diagnostic abdominal MR imaging on a prototype low-field 0.55 T scanner operating at two different gradient strengths. Abdom Radiol 2021;1:3. 10.1007/s00261-021-03234-1. PMC8720489 34415411

[23] Keras Chollet F. 2015.

[24] Abadi M, Barham P, Chen J, Chen Z, Davis A, Dean J, Tensorflow: a system for large-scale machine learning. USENIX Symp Oper Syst Des Implement 2016: 265–83.

[25] Abadi M, Agarwal A, Barham P, Brevdo E, Chen Z, Citro C, Tensorflow: Large-scale machine learning on heterogeneous distributed systems. arXiv preprint 2016: arXiv:1603.04467.

[26] Huang G, Liu Z, van der Maaten L, Weinberger KQ. Densely Connected Convolutional Networks. Proceedings of the IEEE conference on computer vision and pattern recognition; 2016. p. 4700–8.

[27] Rajpurkar P, Irvin J, Zhu K, Yang B, Mehta H, Duan T, CheXNet: Radiologist-Level Pneumonia Detection on Chest X-Rays with Deep Learning. arXiv preprint; 2017, arXiv:1711.05225.

[28] Deng J, Dong W, Socher R, Li L-J, Li Kai, Fei-Fei Li. ImageNet: A large-scale hierarchical image database. Inst Elect Electron Eng (IEEE) 2010:248–55. 10.1109/cvpr.2009.5206848.

[29] Dozat T Incorporating Nesterov momentum into Adam. ICLR Work 2016;1: 2013–6.

[30] Robin X, Turck N, Hainard A, Tiberti N, Lisacek F, Sanchez JC, pROC: an open-source package for R and S+ to analyze and compare ROC curves. BMC Bioinform 2011;12:77. 10.1186/1471-2105-12-77. PMC3068975 21414208

[31] Zhou B, Khosla A, Lapedriza A, Oliva A, Torralba A. Learning deep features for discriminative localization. In: Proc. IEEE Comput. Soc. Conf. Comput. Vis. Pattern Recognit., vol. 2016- Decem. IEEE Computer Society; 2016. p. 2921–9. 10.1109/CVPR.2016.319.

[32] Grøvik E, Yi D, Iv M, Tong E, Rubin D, Zaharchuk G. Deep learning enables automatic detection and segmentation of brain metastases on multisequence MRI. J Magn Reson Imaging 2020;51:175–82. 10.1002/jmri.26766. 31050074 PMC7199496

[33] Tandel GS, Biswas M, Kakde OG, Tiwari A, Suri HS, Turk M, A review on a deep learning perspective in brain cancer classification. Cancers 2019;11(1). 10.3390/cancers11010111. PMC6356431 30669406

[34] Yoo Y, Tang LYW, Brosch T, Li DKB, Kolind S, Vavasour I, Deep learning of joint myelin and T1w MRI features in normal-appearing brain tissue to distinguish between multiple sclerosis patients and healthy controls. NeuroImage Clin 2018; 17:169–78. 10.1016/j.nicl.2017.10.015. 29071211 PMC5651626

[35] Smith-Bindman R, Miglioretti DL, Larson EB. Rising use of diagnostic medical imaging in a large integrated health system. Health Aff 2008;27:1491–502. 10.1377/hlthaff.27.6.1491. PMC2765780 18997204

[36] Abadi E, Segars WP, Tsui BMW, Kinahan PE, Bottenus N, Frangi AF, Virtual clinical trials in medical imaging: a review. J Med Imaging 2020;7:1. 10.1117/1.jmi.7.4.042805. PMC7148435 32313817

[37] Barufaldi B, Bakic PR, Higginbotham D, Maidment ADA. OpenVCT: a GPU-accelerated virtual clinical trial pipeline for mammography and digital breast tomosynthesis. In: Chen G-H, Lo JY, Gilat Schmidt T, editors. Med. Imaging 2018 Phys. Med. Imaging. Vol. 10573. SPIE; 2018. p. 194. 10.1117/12.2294935.

[38] Wagenaar JB, Brinkmann BH, Ives Z, Worrell GA, Litt B. A multimodal platform for cloud-based collaborative research. Int IEEE/EMBS Conf Neural Eng NER 2013: 1386–9. 10.1109/NER.2013.6696201.

[39] Baldassano SN, Brinkmann BH, Ung H, Blevins T, Conrad EC, Leyde K, Crowdsourcing seizure detection: algorithm development and validation on human implanted device recordings. Brain 2017;140:1680–91. 10.1093/brain/awx098. 28459961 PMC6075622

[40] Kohli MD, Summers RM, Geis JR. Medical image data and datasets in the era of machine learning—whitepaper from the 2016 C-MIMI meeting dataset session. J Digit Imaging 2017;30:392–9. 10.1007/s10278-017-9976-3. 28516233 PMC5537092

[41] Greenspan H, Van Ginneken B, Summers RM. Guest editorial deep learning in medical imaging: overview and future promise of an exciting new technique. IEEE Trans Med Imaging 2016;35:1153–9. 10.1109/TMI.2016.2553401.

[42] Taylor AJ, Salerno M, Dharmakumar R, Jerosch-Herold M. T1 mapping basic techniques and clinical applications. JACC Cardiovasc Imaging 2016;9:67–81. 10.1016/j.jcmg.2015.11.005. 26762877

[43] Blystad I, Warntjes JBM, Smedby O, Landtblom A-M, Lundberg P, Larsson E-M. Synthetic MRI of the brain in a clinical setting. Acta Radiol 2012;53:1158–63. 10.1258/ar.2012.120195. 23024181

[44] Wu Z, Chen W, Nayak KS. Minimum field strength simulator for proton density weighted MRI. PLoS One 2016;11:e0154711. 10.1371/journal.pone.0154711. 27136334 PMC4852924

[45] Yi X, Walia E, Babyn P. Generative adversarial network in medical imaging: a review. Med Image Anal 2019;58:101552. 10.1016/J.MEDIA.2019.101552. 31521965

[46] Glasser MF, Sotiropoulos SN, Wilson JA, Coalson TS, Fischl B, Andersson JL, The minimal preprocessing pipelines for the human connectome project. Neuroimage 2013;80:105–24. 10.1016/j.neuroimage.2013.04.127. 23668970 PMC3720813

[47] Dewey BE, Zhao C, Reinhold JC, Carass A, Fitzgerald KC, Sotirchos ES, DeepHarmony: a deep learning approach to contrast harmonization across scanner changes. Magn Reson Imaging 2019;64:160–70. 10.1016/J.MRI.2019.05.041. 31301354 PMC6874910

[48] Cetin Karayumak S, Bouix S, Ning L, James A, Crow T, Shenton M, Retrospective harmonization of multi-site diffusion MRI data acquired with different acquisition parameters. Neuroimage 2019;184:180–200. 10.1016/j.neuroimage.2018.08.073. 30205206 PMC6230479

[49] Mirzaalian H, Ning L, Savadjiev P, Pasternak O, Bouix S, Michailovich O, Inter-site and inter-scanner diffusion MRI data harmonization. Neuroimage 2016; 135:311–23. 10.1016/j.neuroimage.2016.04.041. 27138209 PMC5367052

