# Supplementary material for: Simulated diagnostic performance of low-field MRI: Harnessing open-access datasets to evaluate novel devices
Source: Magn Reson Imaging. Author manuscript; Available in PMC 2022 Apr 1. (PMC8816889; doi:10.1016/j.mri.2021.12.007)
Supplement: 1 [file NIHMS1769415-supplement-1.docx]

**Simulated Diagnostic Performance of Low-Field MRI: Harnessing Open-Access Datasets to Evaluate Novel Devices**

**Supplemental Materials**

**
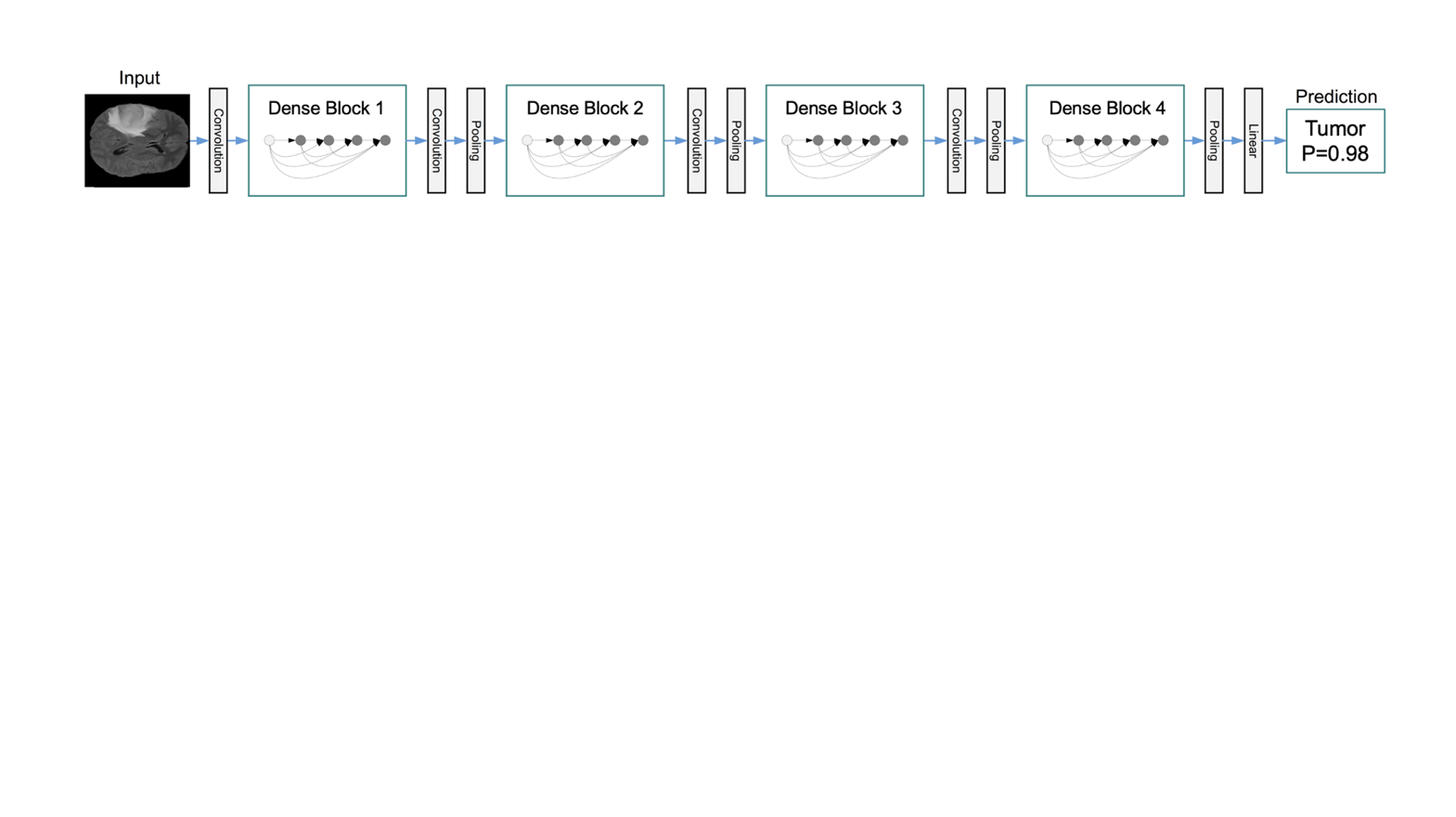
**

**Supplemental Figure 1:** Model architecture. Input to the model is a single axial MR image (224 x 224 pixels). The model consists of 4 dense blocks separated by transitional blocks containing convolutional and pooling layers. Details of the computational blocks can be found in the original paper by Huang, et al., at <https://arxiv.org/abs/1608.06993>. The output is a binary classification (lesion versus no lesion) with a prediction probability.


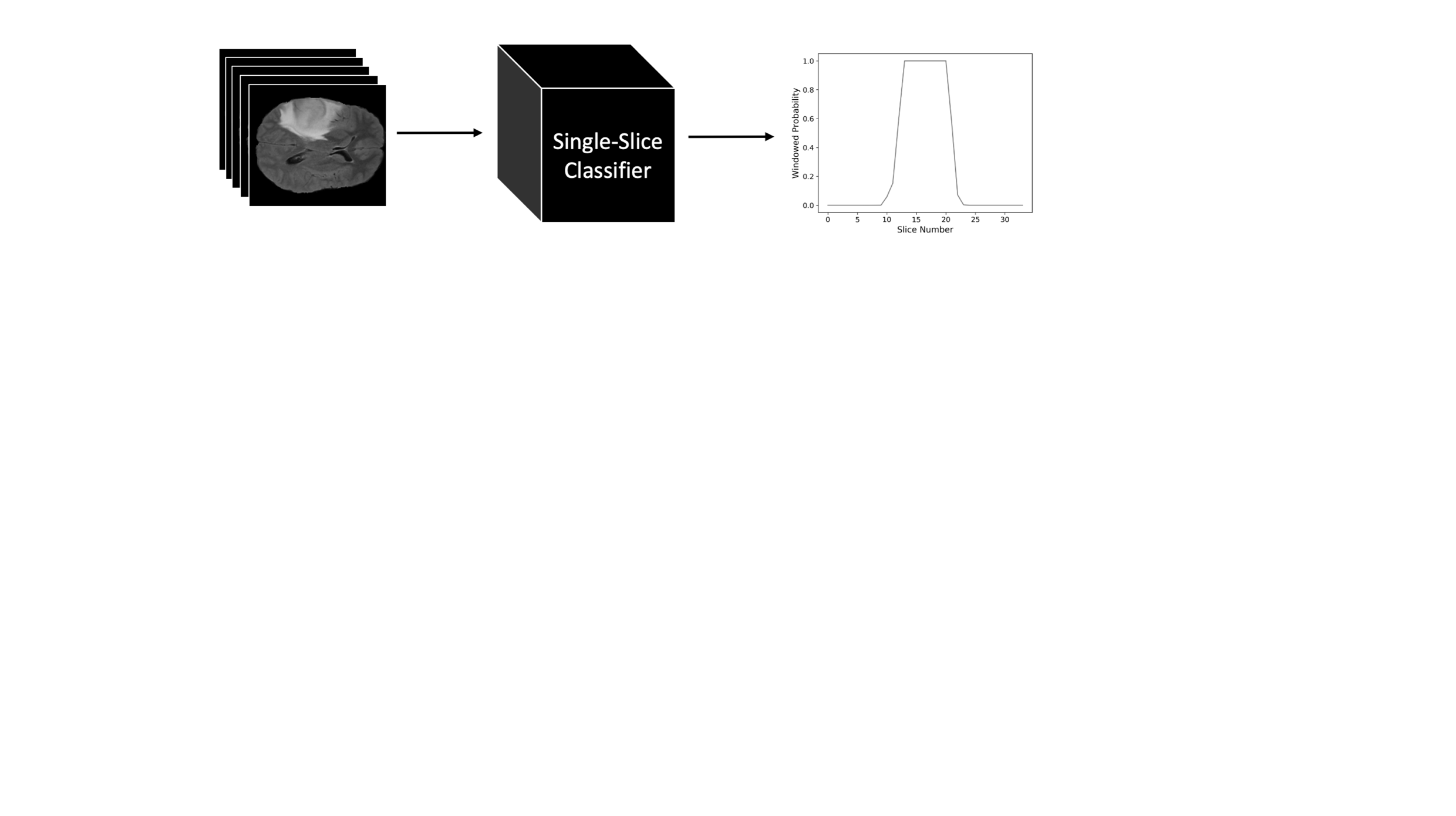


**Supplemental Figure 2:** Subject-level classification. The subject-level classifier builds on the single-slice classifier. Each axial slice in a subject’s image is classified using the single-slice classifier to generate a predicted lesion probability. A convolutional filter (incorporating approximately 1.5cm) is passed over the contiguous prediction probabilities to generate a locally windowed average. If the convolutional filter outputs a probability above a specified threshold, then the subject is classified as containing pathology.

**
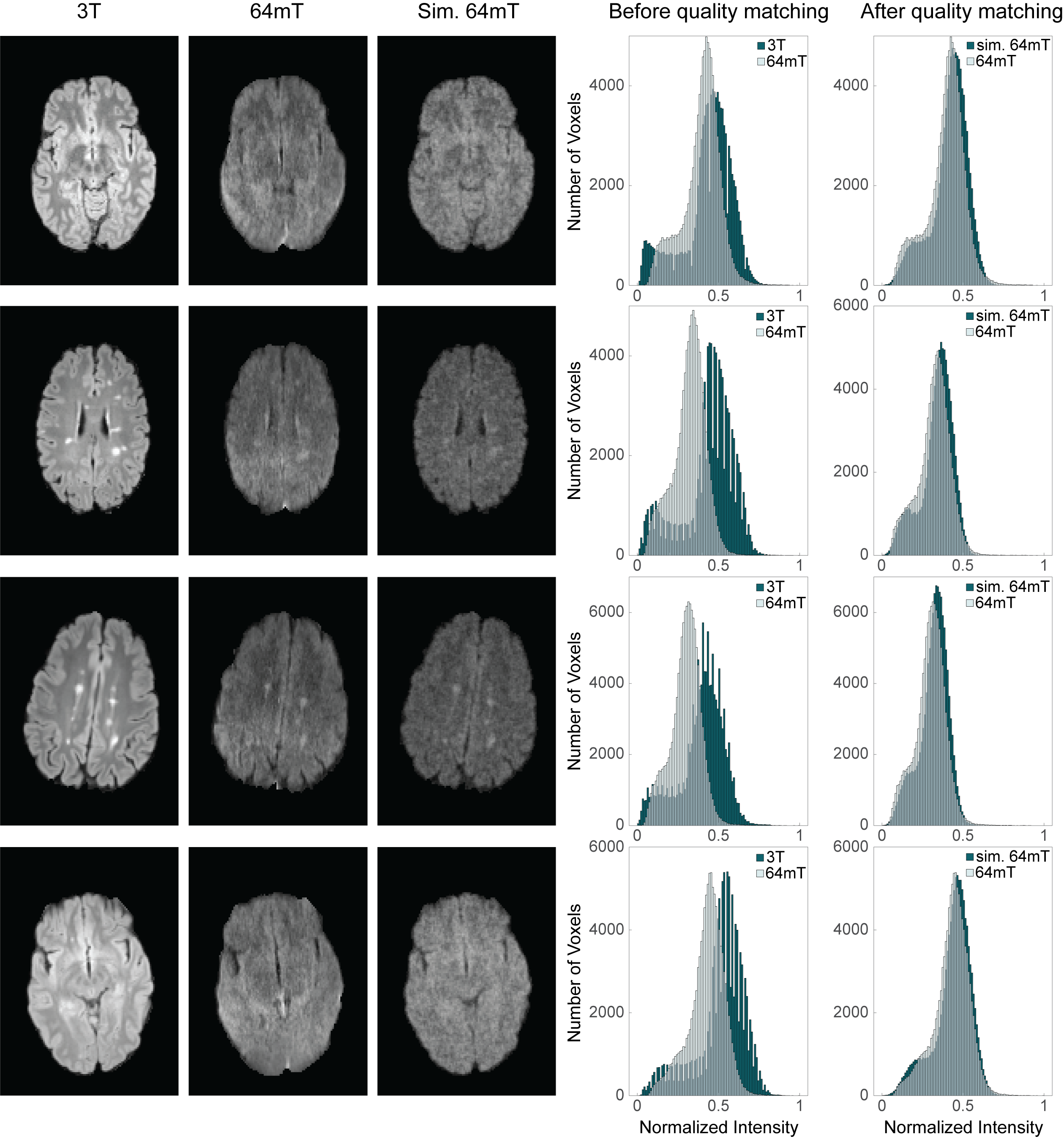
**

**Supplemental Figure 3**: Transformation method applied to novel subjects. Each row corresponds to a patient with multiple sclerosis. The first and second column show the patient’s paired 3T and 64mT images collected from the respective scanners. The 3T imaging has been coregistered to 64mT imaging and resliced for histogram comparison. The third column shows the simulated 64mT image that was generated using the 3T image and the previously developed transformation model. The fourth column compares histograms between the resliced 3T and real 64mT imaging. The fifth column highlights histogram similarity between real and simulated 64mT imaging after transformation.

| **Dataset** | **Num Training Images** | **Num Validation Images** |
| --- | --- | --- |
| LGG, 3T | 10,230 | 1,085 |
| LGG, low-field | 2,376 | 252 |
| HGG, 3T | 35,883 | 3,797 |
| HGG, low-field | 8,334 | 882 |
| MS, 3T | 10,824 | 1,428 |
| MS, low-field | 812 | 124 |
| Stroke, 3T | 2,553 | 614 |
| Stroke, low-field | 865 | 143 |

**Supplemental Table 1:** Training and validation split. Each dataset was split into training and validation sets with an approximate 9:1 division. A total of 8 classifiers were trained (4 pathologies, with separate 3T and simulated low-field classifiers). Abbreviations: High Grade Glioma (HGG), Low Grade Glioma (LGG), Multiple-Sclerosis (MS).
